# First District Dental Society, State of New York

**Published:** 1886-01

**Authors:** 


ARTICLE II.

### FIRST DISTRICT DENTAL SOCIETY, STATE OF NEW YORK.

The First District Dental Society of the State of New York held a special meeting, Tuesday evening, September 22, 1885, in the rooms of the S. S. White Dental Mfg. Co., Broadway and Thirty-second street.

The president, Dr. William Carr, in the chair.

### Incidents of Office Practice.

Dr. B. C. Nash.—I have here a cast of the mouth of a patient, a young lady fourteen years of age, showing an unusual retention of temporary teeth and backwardness in the development of the permanent ones. In the upper arch are the permanent central incisors, bicuspids, and first molars; the lateral incisors and cuspids of the temporary set are retained, and the second permanent molars are still undeveloped. In the lower arch the six permanent front teeth are in position and also the first permanent molars, and on one side a second molar, but there is no indication of the appearance of the bicuspids, though the temporary molars on one side became loosened and were extracted about a year ago. The permanent teeth are generally of excellent quality, but much smaller than usual. I would like advice in this case, as I have heard of gentlemen who invariably extract temporary teeth when the time for their shedding comes. I have not extracted any of these teeth, and am inclined to await developments before doing anything in the matter.

(The opinions given were in confirmation of this view, and the matter was passed.)

DR. W. H. ATKINSON.—Mr. President and gentlemen, I am about to present to you a paper which deals with a disputed point. I wish to invite your close attention, and I put you at liberty to stop me at a comma, semicolon, colon, period, or paragraph; and if I use a term that does not strike you as being relevant, stop me right there.

Dr. Atkinson then read the paper entitled

### PYORRHŒA ALVEOLARIS.

"Flow of pus from the tooth-sockets" has long been recognized as a disease which it was impossible or difficult to successfully treat. Those who have dealt with it are divided as to the character of the departure from health, and as to the manner of treatment. Dr. Riggs regards it as a local disease, and amenable to local surgical cure; while many others, mostly homeopathists, attribute it to constitutional cachexy; and still others regard it as localization of a systemic debility. My own view is coincident with the latter.

The great merit of Dr. Riggs' practice is in his insistence upon the necessity for thoroughly removing *all* the altered tissues well down into the healthy structure, thus favoring reproduction by "first intention." His demerit is his persistent denial of any utility in constitutional or local medication.

All diversity of opinion respecting the diagnosis and treatment of this disease, and in fact of all diseases, lies in our tarrying in mere opinions, and acting as if they were well-established knowledge of nutrition and its aberrations. The various tissues of which the organs of the body are composed have characteristic degrees of form, density, toughness, pliability, and extensibility, by which we are able to distinguish and describe them. All the tissues are supported by the process known as nutrition. Hard tissues feed slowly; soft tissues are more rapid feeders. Hence nutrient changes differ in facility and rapidity in the various tissues in health, as well as in difficulty and slowness in the ratio of departure from the protoplasmic state, in which the most rapid nutrient changes occur.

The bottom facts of nutritional changes are so occult, in consequence of the smallness of the bodies in which they take place, that few have the patience and earnestness to study them for themselves ; and therefore nearly all the recorded information on this subject is a repetition of the crude mass-observations of beginners in this field of research which have found their way into text-books and journals, so lacking in coherence of statement as to make it difficult for any pupil to master them so as to give to them a hearty assent by comprehending the changes described, or to reject them as non-understandable.

Nutritional changes consist of play of affinities so fine and evanescent as to defy formulation in such coarse terms as abound in the books recording the apprehensions of those who have made them a study. Hence the prevalence of adverse estimates pronounced in such cases on the one hand, and of enthusiastic assertions of easy and simple cure resulting in "all" cases with only "one application" of the "remedy" on the other hand. I have seen so many cases of the latter class, which had been pronounced cured with "one thorough treatment,"—in which, by the way, there were teeth which were not only loose in the sockets, but which had fistulæ which were discharging more or less of the broken-down tissue,—that I feel bound to raise a warning voice against unwise confidence in the short, sharp, and quick method of treatment. Not that there are no cases so cured, but that so many supposed to be cured have proved to be only palliated and set back, to break out again when they were lost sight of by the operator who had pronounced them cured, to fall into, sometimes, less competent hands.

Under these experiences we are led to suppose that different conditions have been grouped together as being the same form of disease, or that differing degrees of degeneration have been deemed to be alike amenable to one single remedy applied in one way, and that "my way."

I am convinced that we will not see eye to eye until these cases shall have been made subject to clinical investigation and demonstration such as has had marked success in the other branches of our art, viz., filling teeth and fitting regulating and restorative fixtures, now so far advanced toward perfection.

One of the greatest obstacles in the way of understanding the various modes of nutritional change is the classification by which those which belong together are named as if apart and distinct. All nutritional metamorphosis occurs in protoplasmic bodies, which are the elements of every form of nutrifying body which converts pabulum into tissue. Where protoplasm is not formed into tissual limitations, of neural, osseous, muscular, connective, or epithelial differentiations, the ameboid form of feeding holds the dominion of the field of nutrient activity, which in reality is the only condition in which these changes can take place. Therefore all foods must be reduced to the fluid state before appropriation is possible.

Atrophic dyspepsia of connective-tissue is the most difficult of detection, and is the first step in every case of pyorrhœa alveolaris.

The bond of union between the tooth and its socket consists of a connective-tissue layer attaching it on its inner border with the cement corpuscles, and on its outer border with the myxomatous tissue of the gum, which in turn is covered with an epithelial coat consisting of several layers of epithelial bodies of globular, cuboidal, cylindrical, and squamous conformations. The cement-substance connecting these elements of the tissues is nitrogenized hyperoxidized hydrate of carbon. In other words, the ameboid ectosarc is of such plasticity as readily to hold to or let go of its fellows composing protoplasm, mucus, blood, muscle, nerve, or epithelium.

To this fineness of interpretation, then, do we come at last to enable us to catch a glimpse of the territory in which the first divergence of health is displayed in every case of "pyorrhœa alveolaris."

Wasting of the cement releases the hold of the connection, which by the resilience of gum-tissue opens a gap at the point of death of ectoblastic structure. In this chasm various deposits may occur. Where inflammation is induced, it may resolve or proceed to suppuration or sphacelus, caries or necrosis, according to the constitution and status of health of the body at the time. As a rule, the earlier and more manageable stages of this disease are not noticed by either the patient or the practitioner, and hence well-pronounced cases are those which generally apply for relief.

When a ripened germ is separated from the bed in which it was generated, the act may well be named "parturition." If, then, protoplasm be the first form in which the feeding process can be proved to occur, are we not justified in the assertion that rejection of excess of food and incompatiple material is the first example of increment and decrement, of coming together and going apart, or impregnation and parturition of elemental bodies?

All the functioning bodies of which we have any knowledge are composed of elemental bodies too small to be seen. When any department is denied its normal demand for nutrition, the harmony of function is lost, and career of body thus minified is cut short or destroyed. When enough destruction has been effected to be detected, the case is ready for investigation, diagnosis, prognosis, and treatment, or abandonment.

Concretions of lime are never causal of disease; only concomitant or sequential. No two specimens of calcareous deposit have yielded an identical analysis. They are the result of a breaking down of tissue-elements under stress of disease in which the acids requisite to holding the lime in solution are deficient in supply, or brought into contact with bases for which they hold higher affinity. The attempts to classify these deposits have resulted in a somewhat ambiguous nomenclature, viz., salivary calculus, serumal calculus, and sanguinary calculus.

The general term in common use to designate these deposits is "tartar," originating in a loose resemblance to the crust on wine casks called by this term.

Whenever a deposit is formed it takes the shape of the pocket in which it is precipitated from the fluid holding it in suspension. Take the example of the pocket produced by the recession of gum about the neck of the tooth from solution of the dental ligament, and we find the deposit to conform to the shape and size of the pocket, from a mere nodule to the segment of a circle, or to an entire ring about the neck of the tooth. Take a case of the so-called serumal or sanguineous deposit at the end, or near the end, of the root, where the solution of the connective-tissue corpuscles forming the pericemental tract has broken down the cement-substance which fuses the corpuscles into a sheet or membrane, and we find the nodules to correspond to the chasm formed by the retrogressive nutrient act in form and extent of the separated tissue. All calcareous deposits in an inclosed chamber necessarily arise from the precipitation of the lime in the locality, and must be from the circulation direct, or the broken down molecules and corpuscles of the tissues in the neighborhood. Deposits in open chambers may be from mucus, saliva, or free solutions of lime-salts, and may be properly named when we are able to determine their place of formation. The irregular composite calcareous bodies found in the respiratory, genito-urinal, and other circulatory tracts are easily understood so soon as their habitat is determined. The great masses of lime found in the lungs, the kidneys, the liver, the joints, and the mouth are all very interesting subjects of study, and are developed under the general laws of nutrition by organic, tissual, corpuscular, and molecular chemistry, and are amenable to classification when their origin is ascertained.

If a calculus contains cholesterine, we refer it to the liver for origin; if carbonate of lime predominates, we refer it to the respiratory tract; if phosphates and urates are present, to the urinal apparatus, if the carbonates and phosphates be mixed with epithelia and heterogenous foreign bodies, it is referred to the mucous and salivary tracts for origin. Wharton's duct, Steno's duct, and the necks of the teeth are the places where we may find concretions of this last character, as instance the large masses so often found about teeth which are not vigorously used.

If what has been said be comprehended, there remains only a very short statement to complete this paper upon pyorrhœa alveolaris.

The three degrees or stages of this affection must be met by three degrees of extirpative energy: 1st, physiological; 2d, mechanical; 3d, chemical.

The first includes good feeding and hygienic cleanliness; the second, removal of foreign material by mechanical means; and the third, the destruction of ferments and their results by such means as kill the spores and their products and the debilitated tissue-elements, so that the physiological activities may throw off the offensive and effete matter and reproduce the tissues normal to the location.

To speak of the details of these methods of cure, and set forth their claims to attention, would involve the presentment of cases in the various stages and the special remedy to be resorted to in each. To give a mere hint of this labor, the best I can do is to give a former classification of application and remedy.

Where slight loss of the border of the gum is present, elixir of vitriol is the proper application to effect the purpose of inciting a return of physiological activity. Where greater loss of connection between tooth and socket is present, with some lime deposit, use a solution of aqua regia, one part to seven of water. In cases of greater loss of attachment and loss of considerable portions of the alveolar plates, with or without foreign deposits, use caustic paste, made by melting together caustic potash ("potassa fusa") and crystalized carbolic acid, so as to make a homogenous paste, which upon cooling will be a solid and coherent mass, capable of being broken into bits of such size as to meet the demands of each case. Place these bits upon the site where you wish to form the eschar down to normal growth. The warmth will melt them and allow the affinities between this remedy and the altered tissues to convert all the dead and dying parts into a scab or eschar, which will form the limit of the pocket where sloughing occurs, into which the new protoplasmic exudate will form the clot out of which to secure the new growth. Wherever the parts press upon the locality so as to prevent or displace the exudate, a fixture must be resorted to to secure the clot in place long enough to enable it to be metamorphosed into the tissues normal to the part, from protoplasm (the clot) to embryonal corpuscles, myxomatous, connective, neural, vascular, and epithelial tissues, beneath which the new osseous growths will reproduce the sockets of the teeth.

DISCUSSION.

DR. FRANK ABBOTT.—I wish to congratulate Dr. Atkinson upon the excellent paper he has presented to us. While I differ with him upon some points in treatment, still I am sure he has given us most valuable thoughts for consideration. There are some parts of it, perhaps, that few of us understand clearly, and it may take us many years to get to the point of understanding them as he does. He commences his paper with Dr. Riggs' treatment of this disease, which is known among dentists, to some extent as "Riggs' disease." To me this is a very unsatisfactory name, and I never use it. There are so many ways of treating the different phases of this disease that it would be necessary to present cases in order to come to any definlte conclusion as to what we can and should do in any particular case. As for the surgical treatment, properly speaking, as practiced by Dr. Riggs, I believe it is too severe. I do not think there is any more necessity for cutting into the soft tissues, or attempting to cut away any portion of the alveolus, than there is for cutting into the gum outside or the tissues in any other location in the mouth. I have never yet seen a case in which the alveolus could be reached with an instrument without first cutting through the soft tissue. That Dr. Riggs does cure some cases of this disease by local treatment is probably true, for there are cases where local treatment is all-sufficient, and Dr. Riggs, or any other man, if he thoroughly cleanses the teeth from the deposits about them, will frequently get a return to health without any medication. But this will not hold good in all cases. In the cases I have had under treatment I have found no necessity for using sulphuric acid, aqua regia, or caustic potash, for the reason that, although I have not cured, I have relieved every case that I have had to do with without such severe treatment. That an absolute cure can be effected with the above-named remedies, leaving no necessity for further treatment at any subsequent time, is in my opinion claiming altogether too much. *Pyorrhœa alveolaris is never cured,—i. e.,* the normal conditions of the parts restored. That it can be relieved for the time being, and may appear to be cured, I know is true; but the same condition that first developed the disease still remains in the patient's system, notwithstanding the treatment, particularly if it be altogether local. Now, the same primary conditions existing, the same results will recur unless the case is followed up and the treatment repeated every few months, certainly as often as once or twice a year.

I have heard it stated that cases of pyorrhœa alveolaris occur without any deposit whatever upon the teeth, and also that this disease was entirely and emphatically due to some constitutional taint which produced the disturbance in the gum and interfered with its nutrition, causing the gum to detach itself from the necks of the teeth and leave an open pocket, without having, however, any deposit around the roots of the teeth, either calculary, serumal, or sanguinary. *There is some deposit on the teeth in every instance that I have ever seen.*

The doctor spoke of the pericementum or membrane attaching the root of the tooth to the parts adjoining. I heard this last summer quite a labored paper, taking forty-five minutes or more in the reading, and very vigorously read, too, in which it was attempted to establish what was claimed to be a fact that there were two membranes surrounding the root of a tooth. This, I believe, from actual observation under the most powerful microscopes, has never been proved. Possibly I may make a mistake, but I have never yet seen it, although I have examined this membrane many times under the microscope, and those who are best acquainted with that tissue, and who have studied it longest and most carefully, are of the same opinion. The paper I refer to was read at the meeting of the American Dental Association at Minneapolis, and, I think was not discused at all.

From one statement made by Dr. Atkinson, as I under stood it, I got the impression that he wished to imply that in pyorrhœa alveolaris the alveolus surrounding the root of a tooth commences first to "waste away;" that the gum, in consequence, loses its attachment to the neck of the tooth, leaving an open pocket. Possibly that may be the correct idea, but my impression has always been that the gum first became inflamed to such an extent that its nutrition was interfered with; want of nourishment caused it to detach itself from the neck of the tooth, and thus a pocket was formed. This is what occurs in cases of salivation. The gums become diseased because nutrition is interfered with, and their attachment to the necks of the teeth is destroyed. I have heard the statement that no case of pyorrhœa alveolaris ever occurred except where the patient had been salivated. Whether this is so or not I am not ready to state, but I believe that every person who has been salivated suffers more or less from this disease. I have been of the opinion, and am still, that under the irritation caused by deposits at the margin of the gum the pericementum throws out lime salts which, mixed with epithelial scales and particles of food, are deposited upon the roots of the teeth, forming what we call tartar. In my treatment of all cases of this kind I have depended almost entirely upon a careful use of my instruments and the perfect removal of the deposits from the teeth, making them as smooth as I could, and simply washing out the pockets with a weak solution of carbolic acid. I believe that by this treatment I can accomplish all that can be accomplished in any way with a disease of this kind.

DR. J. L. WILLIAMS.—I don't think that I can add anything to the remarks that have been made; and it is pretty difficult to add anything to the aphoristic statements of Dr. Atkinson. If I were to say anything at all it would be in the line of a kindly criticism of the remarks of that gentleman, with an interrogation point at the close.

It seems to me that there is a great deal of difference of opinion and mystery hanging around the real nature of the trouble itself; and the treatment, of course, differs according to the conception of what the trouble is in its origin,—whether it is purely local, which it seems to me is a contradiction in terms, or whether it is a local expression of a constitutional disturbance. The view that is taken of the disease will determine the treatment. If it is a purely local trouble, surgical treatment is all-sufficient. But it can hardly be that, even if the immediately antecedent deposit of lime-salts around the teeth were sanguinary or salivary, because even that deposit indicates a perverted physiological condition somewhere in the body. If, as Dr. Atkinson says, the immediate antecedent of pyorrhœa be atrophic dyspepsia of the connective-tissue elements, then it must be a local expression of a constitutional disturbance. If that is true, that it is a local expression of a constitutional disturbance, what right have we to expect local treatment alone, whether it be surgical or medical, or both combined, to prove more than palliative? Of course, the progress of the disease in most cases is slow. The advanced cases have been a long time in coming to the condition in which they are found, and a purely local treatment, either surgical or medical, will for a long time palliate the difficulty; but if the disease is a local expression of a constitutional disturbance, then it seems to me that such treatment must be purely palliative, and that it is not scientific to say that a cure has been effected by it. If the treatment springs from some constitutional disturbance, is it not sure to return unless that constitutional disturbance be corrected?

DR. ABBOTT.—I did not make any statement in reference to what I believed the disease consisted of; but I do believe it is a local expression of a constitutional disturbance, just as the reader of the paper does. I have not any doubt that judicious constitutional treatment might do a great deal towards relieving these cases. What that constitutional treatment is or should be is a question that we have yet to determine positively. I would like to ask Dr. Atkinson what constitutional treatment he adopts in such cases, if any.

DR. ATKINSON.—I almost invarably use constitutional treatment. It is only those cases that are very well endowed with good blood crasis that do not need constitutional treatment. Nutrition is the only means of cure. That goes without saying. That is physiological and pathological common sense. We know that everything that feeds must have something to feed upon, and if it feeds upon something in order to live, that which is called life must be transferred from the food to the feeder.

DR. ABBOTT says he understood me to say that it was the alveolar process that was first involved in this disease. If he will read the paper when it comes to be published, if it shall be accorded that honor, he will see that I said atrophic dyspepsia of the connective-tissue was the first step. I mean—not the cementum, which we say constitutes the modification of the bone that makes the connection around the teeth—I mean the estosarc, or outside skin of the ameboid body which we call the connective-tissue corpuscles, and which makes the lining of the socket and the tendons of the body. It has that peculiar waxy surface that enables those elements to be fused together, and that constitutes the tendons and the membranes. It is precisely the same as when you get a hang-nail; the first point that you tear away breaks the connection and leaves a hanging part. The reason why we don't understand these expressions of disease is that we have been looking at a mass of tissues in organs and not at the molecules which constitute them.

There is not a man in medical practice that is worth a snap to give advice in these cases. I saw that in one fresh from school at the Minneapolis meeting. Western men, although they follow the old text-books, are willing to learn and to ask assistance from whatever source they can get it. They take us to be worth something, and they invite us to examine their cases, but we found them still pursuing the treatment laid down in the books, and following what was given up as effect years ago. They were poulticing periostitis of the jaws and alveolar abscseses.

I want to fasten upon your minds the growing necessity for the dentists of this city to establish a free hospital into which everyone who is suffering shall be able to go and receive intelligent treatment, where we shall demonstrate the very best ability of the profession, and join in having such clinics as we have never yet had; where we shall have such diagnosis, treatment, and cure as have never yet found pronouncement on this planet. When we shall have such an institution as I hope will be etablished in this city, so that we can have those things brought before the minds of all and get the best light on them, where we can pool our issues and let every man have the benefit of the best ability and work, then we will make a splendid advance. I wish I had time to make the demonstrations as clear to your minds as they are to my own. It is the nutrition of the five tissues that constitute the human body that we must understand, and know how the food we eat is converted into pabulum, how that pabulum is converted into blood, how the blood is transformed into protoplasm, and protoplasm into embryonal corpuscles, and embryonal corpuscles into the various tissues that constitute the organs of the beautiful machine that we call the human body, and which stands, according to the old Greek word *anthropos*, with upward-turned face.

The treatment for all patients that you will see, with the exception of about one per cent., will be to give a two-grain pill of the sulphate of cinchonidia night and morning. I have some patients who have pursued that treatment for four years. If they are at all nervous, then take McKesson & Robbins' nux vomica, phosphorus and cantharides, one pill each day, in addition to the four grains of cinchonidia. Some patients require a little more, some a little less, but it is not often that they require less than that. Why do we give cinchonidia? I have a suspicion that what we agree to call cruorin, which has a red color and means a red corpuscles of the blood that is carried through the system for its use, is so nearly like the sulphate of cinchonidia that there is no chemist who has been able to show the difference. Hence I take it that this cinchonidia is readily convertible into the cruorin which constitutes the red blood-corpuscles of the blood.

The treatment I have indicated is the general treatment. I can show cases of school-teachers who were all "played out" when they came to me, and who are now in full health and happy. I have named the simple prescriptions that I have given for a long time. There are other prescriptions, such as elixir of vitriol,—half a teaspoonful in a wine-glass of water, to be given at meals to people who are all played out. There is another remedy to which a gentleman in this room, I think, owes his life, and that is aqua regia,—five drops in a glass of sweetened water, or ten drops to be taken after each meal for one or two weeks; then to be repeated after an interval as may be required. Then you get a gastric juice that does its work.

DR. G. W. WELD.—In cases of anemia does the doctor use chloride of iron?

DR. ATKINSON.—Anemia is a deficiency of the blood. I have given you my idea of what cruorin is. That is the element that is most likely to be lacking. Don't fool yourselves any longer with that notion about iron. There is iron enough in every article of food you eat to supply all of that element that can be used in the body. Iron is not incorporated into the body outside of the blood, in which its special purpose is to make a magnet of the red corpuscle and to invite the oxygen to hang about it for transportation throughout the circulation, to effect the purpose of nutrition, which is combustion. Young muscle is light colored; old muscle is a deep color. Beef is dark in color, and veal light; and that is the key to the destruction of the blood-corpuscle in the nourishment of the muscle in which the cruorin is deposited that gives it its deeper color. Of all the multiplicity of remedies that we use ashes are the active principle. What are ashes? Ashes are the oxides of metals and metalloids.

DR. WELD—I presented at the clinic this afternoon a case of lupus erythematosus of the lips, in which there is, as you know, a peculiar functional activity. It is what is sometimes termed scabbing of the lips. The skin peels off every twenty-four hours. There seems to be at times no help for this. Whether this malady be a local expression of a constitutional disturbance or not, I do not know. In this case the patient is forty years of age; he has no scrofula, and no specific taint; he never tasted a glass of liquor in his life, nor did he ever smoke a cigar. He is a powerful man. Evidently his blood contains a great deal of iron, and yet he has been under the care of one of the best New York physicians, who is unable to cure that little disease of the lips, which at least comes under the head of lupus erythematosus. It may not be exactly that, but it resembles it, inasmuch as there is a chronic hyperemia of the parts accompanied by an apparent new cell-growth. Now, what is the difference between this case of chronic disease and one of Riggs' disease? It is simply a want of functional harmony. In one case you have something resembling atrophy, and in the other hypertrophy, and they may be both local expressions of constitutional disease. Regarding the treatment of Riggs' disease, I believe that every intelligent dentist and physician looks to the general health of his patient and prescribes for anemia if that treatment be indicated. Whether you use iron or any other one of the restorative agents for increasing the red blood-corpuscles, if you improve the general health by so doing, you must necessarily relieve the local disease. As far as the mechanical part of the treatment is concerned, I do not think it necessary to say anything. Every gentleman present knows, I think, how to use the instruments and use them skillfully.

DR. ATKINSON.—I saw that case. The doctor says the patient is not of a strumous habit. He has the thick blubber lips that belong to the strumous habit. My impression is that a solution of salicylic acid in alcohol, properly diluted or of full strength, to paint the lips and cook the epithelium so as to make a scab and allow the parts underneath to heal, will work entire cure in that case. That it has any of the characteristics of lupus I did not discover. There was no eating about it. Lupus means a wolf, and all the cases of lupus that I have seen have had a ragged edge as though they were eaten away. They are not very malignant until they have been a long time in the system and have been given opportunity for deteriorating the pabulum that is to be transported to other parts of the system. Adjourned. B. C. NASH, D. D. S., *Secretary*.

—*Dental Cosmos*.

